# The positivity rate of 68Gallium-PSMA-11 ligand PET/CT depends on the serum PSA-value in patients with biochemical recurrence of prostate cancer

**DOI:** 10.18632/oncotarget.27239

**Published:** 2019-10-22

**Authors:** Manuela A. Hoffmann, Hans-Georg Buchholz, Helmut J. Wieler, Thomas Höfner, Jonas Müller-Hübenthal, Ludwin Trampert, Mathias Schreckenberger

**Affiliations:** ^1^ Department of Occupational Health and Safety, Supervisory Center for Radiation Protection, Federal Ministry of Defense, Bonn 53123, Germany; ^2^ Clinic of Nuclear Medicine, Johannes Gutenberg-University, Mainz 55101, Germany; ^3^ Clinic of Nuclear Medicine, Bundeswehr Central Hospital, Koblenz 56072, Germany; ^4^ Clinic of Urology, Johannes Gutenberg-University, Mainz 55101, Germany; ^5^ Practice of Radiology and Nuclear Medicine, Praxis im KölnTriangle, Köln 50679, Germany; ^6^ Clinic of Nuclear Medicine, Klinikum Mutterhaus der Borromäerinnen, Trier 54290, Germany

**Keywords:** positivity rate, 68Gallium-PSMA PET/CT, prostate-specific antigen, prostate cancer, biochemical recurrence

## Abstract

**Background:** The aim of the present study is to analyze the efficacy of 68Gallium (Ga)-PSMA-11 PET/CT for detecting and localizing recurrent prostate carcinoma (PC) in patients with different prostate-specific antigen (PSA), PSA velocity (PSAvel) and doubling time (PSAdt).

**Results:** The PR of 68Ga-PSMA-11 PET/CT showed a positive relationship with PSA levels. Even at restaging PSA-values (PSAV) of lower than 0.2 ng/ml, PR was 41%. For PSAV of 0.2-<0.5 ng/ml the PR was 45%, 62% for PSAV of 0.5-<1.0 and 72% for PSAV of 1.0-<2.0 ng/ml. The PR increased to 85% for PSAV of 2.0-<5.0 and reached 94% at PSAV of ≥5.0 ng/ml. At PSA of <1 ng/ml/y the PR of PSAvel was 50% and increased to 98% at PSA >5 ng/ml/y. No significant association was found for PSAdt.

**Methods:** PET/CT scans of 660 patients with biochemical recurrence (BCR) after primary therapy of PC were included in the analysis. We correlated serum PSA levels, measured at the time of imaging with PSMA PET/CT-positivity rates (PR) as well as PSAvel (in 225 patients) and PSAdt (660 patients). Additionally we compared the incidence of localized disease to metastases as related to these PSA-biomarkers.

**Conclusion:** We have shown, in a large cohort of patients, that 68Ga-PSMA-11 PET/CT is a sensitive tool for restaging PC and has a high detection efficacy, even in patients with very low PSA levels (<0.2 ng/ml). Thus 68Ga-PSMA-11 PET/CT both identify and localize recurrent disease with implications for a more direct treatment approach (localized vs. systemic therapy).

## INTRODUCTION

PC related mortality is almost as high as that of lung cancer and is the most frequent type of cancer in men [1]. Regular screening of PSA levels has shown an incidence of PC of 95–116 per 10,000 person-years and the incidence of death from PC is about 2 per 10,000 person-years [2].

**Figure 1:**
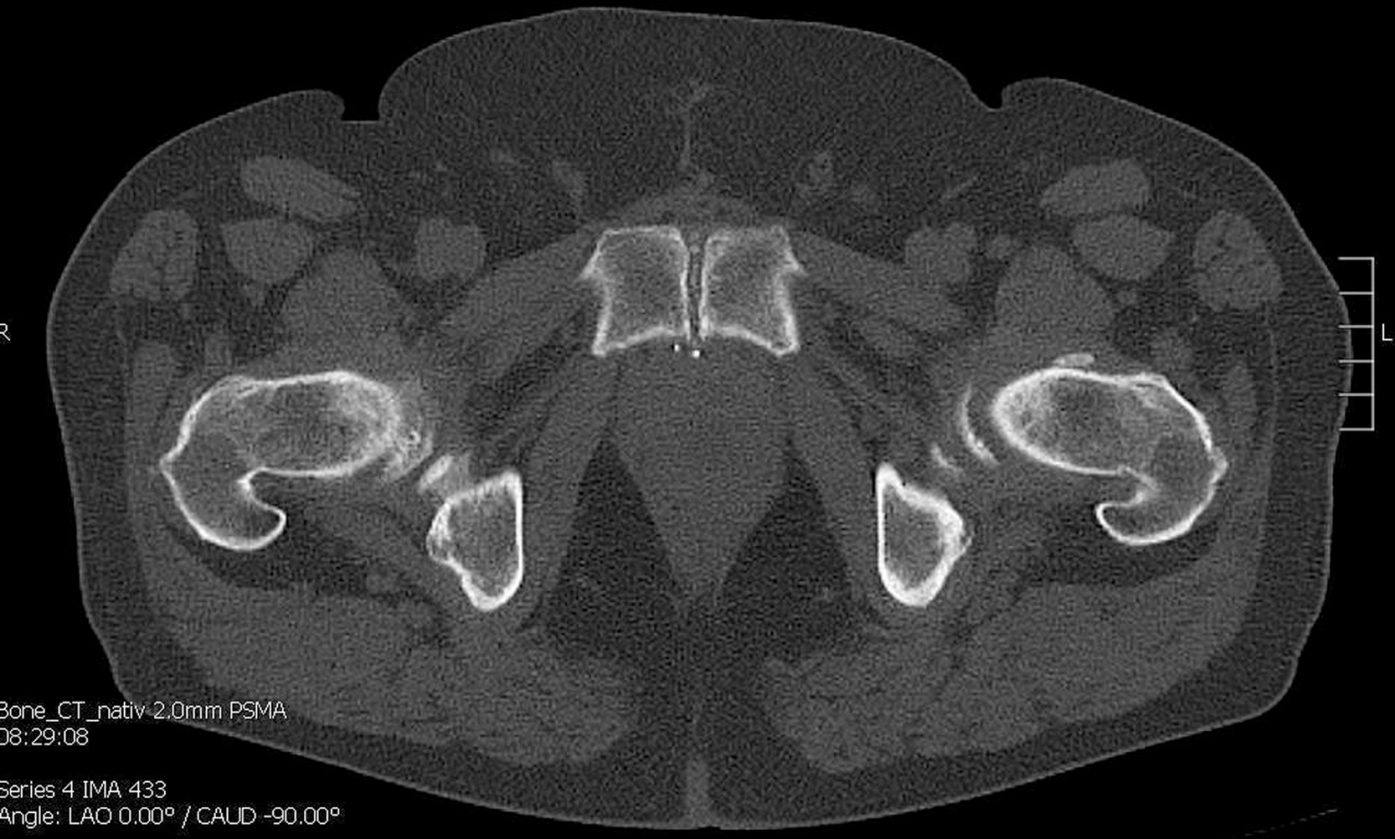
CT shows no bone metastasis in the symphyseal os pubis on the left.

**Figure 2:**
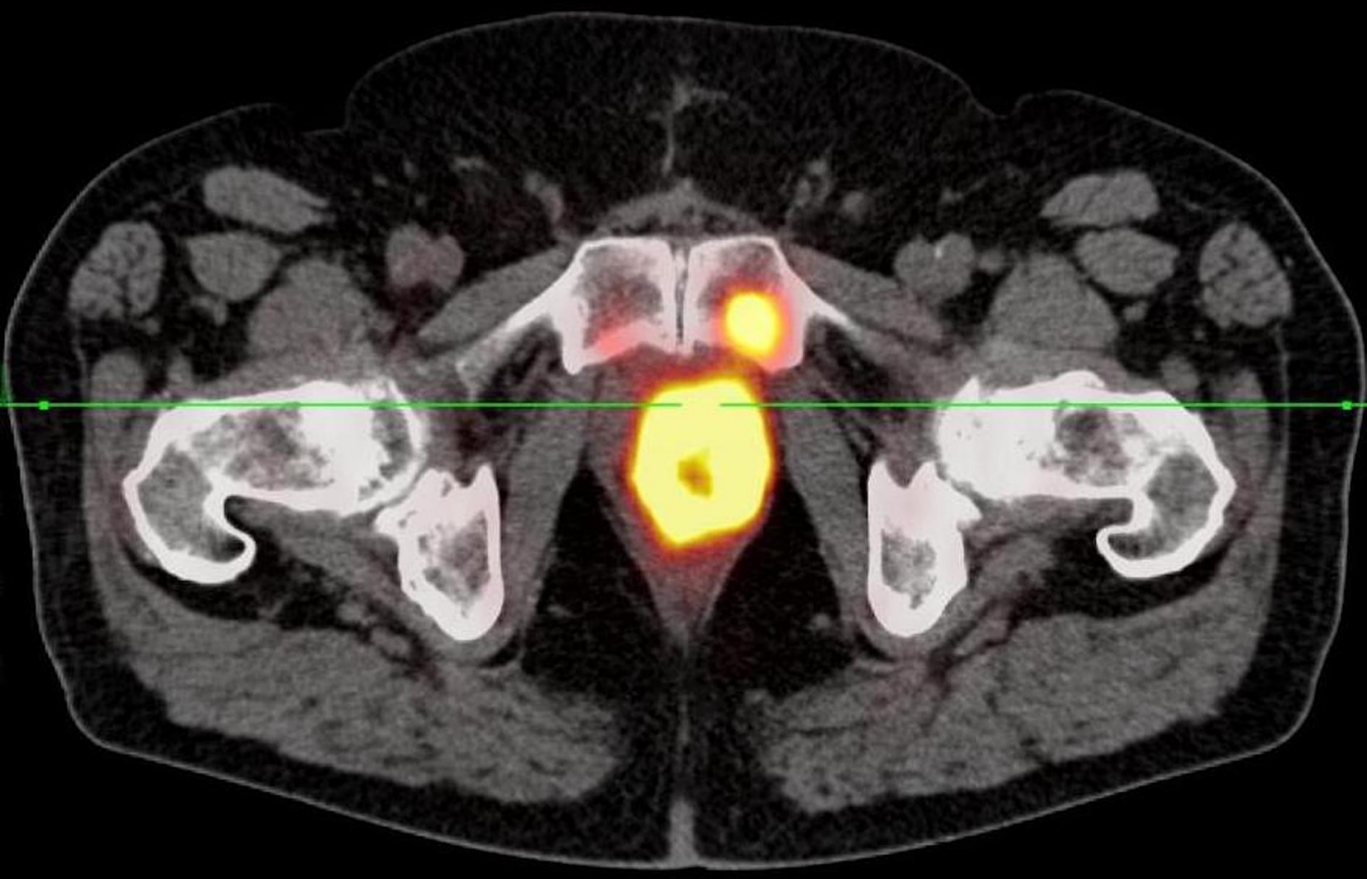
68Ga-PSMA-11 PET/CT shows a bone metastasis in the symphyseal os pubis on the left.

Most patients are diagnosed at an early tumor stage with local disease, for which the primary therapeutic approach is radical prostatectomy or radiation therapy. Depending on the biological behavior and extent of disease, various treatment options are available. Radiation therapy (RT), brachytherapy, external beam RT (EBRT) and intensity modulated RT are commonly used for treating patients with aggressive PC [3, 4]. Despite radical treatment, BCR occurs in about 30% of treated patients [1, 4]. Cancer risk and degree of malignancy can be evaluated by PSA levels and kinetics [3]. When PSA rises to >0.2 ng/ml following radical prostatectomy (RP) or >2 ng/ml above nadir after RT, the definition of biochemical recurrent disease is reached [5]. Above that PSAV, the proportion of patients that will truly develop significant local or systemic recurrent PC increases considerably. If PSA rises ≥0.4 ng/ml after RP with a subsequent elevated value, PSA progression risk is 79% and correlates with the risk of clinical progression. When BCR occurs early, there is a greater risk of metastatic recurrence, and a longer time to BCR is associated with risk for localized recurrence [3]. PSA kinetics such as PSAdt (the time for PSA to increase by 100%) and PSAvel (measurement of how fast PSA increases over time) can be used as prognostic tools in the detection of PC [6]. While PSAdt >12 months suggests the development of local recurrent disease, patients with PSAdt less than 3 months represent a subgroup with a high risk of developing distant metastatic spread [5]. Morphological imaging techniques have limited accuracy in the detection of BCR, e. g. transrectal ultrasound, computed tomography (CT) and magnetic resonance imaging (MRI). Standard imaging techniques for the localization of recurrent disease (99-technetium bone scan, CT scan and MRI) are not very sensitive and it is likely that other diagnostic tests based on metabolic factors (such as PET with various tracers, MR spectroscopy) will be used for the study of BCR after radiotherapy in the near future [7]. Molecular imaging such as hybrid positron-emission tomography (PET)/CT with specific tracers, based on metabolic factors, should improve diagnostic accuracy. Hybrid choline PET can diagnose and localize PC recurrence in patients with low serum PSA levels (1–2 ng/ml) with a PR of 36% [3, 6, 8]. Despite more sensitive detection of PC, 50% of patients develop a second PSA recurrence. BCR-patients scheduled for salvage radiotherapy (SRT) with PSA levels of <0.5 ng/ml have the best long-term prognosis. Therefore, ongoing research is now focused on the development of more sensitive PET molecules to identify BCR at low PSA levels and enable early treatment. Prostate-specific membrane antigen (PSMA) is significantly overexpressed in PC cells, and its expression is generally strong in high tumor aggressiveness, metastatic disease and recurrence of PC, while being weakly or not expressed in benign prostate tissue. Therefore, PSMA is an optimal target for imaging (Figures 1, 2) and therapy of PC [9–11]. HBED-CC or -*N, N’*-bis[2-hydroxy-5-(carboxyethyl)benzyl]ethylenediamine-*N, N’*–diacetic acid is an efficient and stable 68Ga-chelator at low temperature and low concentrations [12]. The small-molecule PSMA inhibitor 68Ga-Glu-urea-Lys (Ahx)-HBED-CC (PSMA-11) has been suggested as a sensitive molecule, for detection of PC and better than choline PET/CT, which is rarely positive in restaging PSA levels under 1 ng/ml [1].

The purpose of this study is to assess the potential of 68Ga-PSMA PET/CT for the detection and localization of recurrent disease in patients with BCR. We aimed to evaluate the dependence of the PR on PSA level and PSA kinetics, and to identify the lowest PSA threshold that detects local, regional or systemic relapse of PC, using 68Ga-PSMA-11 PET/CT. We retrospectively analyzed PSA levels after primary therapy for PC as well as PSA levels at resubmission for BCR in a large cohort of patients taking the Gleason Score (GS is the world’s established PC grading system and used for the histopathological assessment of the glandular morphology of the prostate; since PC is often inconsistent and may have significantly different areas, two areas are generally assessed for GS; biologically significantly more aggressive tumors must be assumed in findings with GS 4+3=7b, 4+4=8, 4+5=9 and 5+4=9 [5, 11, 25]), determined after resection, and anti-androgen therapy **(**AT) into account.

## RESULTS

The main parameters were 68Ga-PSMA-11 PET/CT PR and the association of positive scans with relapse PSA levels, PSAdt, PSAvel as well as the association with different sites of recurrence and the influence of AT.

### Detection efficacy, PSA levels and kinetics

As evidenced by 68Ga-PSMA-11 PET/CT, 500 patients (76%) showed one or more locations suspicious for PC recurrence (Table 1).

**Table 1:** Characteristics of patients and regions and frequency of recurrent PC detected with 68GaPSMA-ligand PET/CT

Characteristics (*n*)	Parameters
Number of patients	660
Age (y) (660)	
- Median	71
- Range	49–88
- Mean ± SD	70 ± 8
Gleason Score (660)	
- ≤ 6 (low risk + grade group 1)	45
−7 (intermediate risk + grade group 2+3)	337
−8 (high risk + grade group 4)	118
- > 8 (high risk + grade group 5)	160
PSA (ng/ml) (660)	
- Median	10.65
- Range	0.1–2000
- Mean ± SD	2.46 ± 95.8
PSAvel (ng/ml/y) (225)	
- Median	1.01
- Range	0–620
- Mean ± SD	9.54 ± 50.74
PSAdt (months) (660)	
- Median	10.65
- Range	0–628
- Mean ± SD	25 ± 52.8
Prior treatment of primary tumor (660)	
- Surgery (radical prostatectomy)	535
- Radiotherapy and other	125
Interval primary treatment - PET/CT	
- Median	41
- Range	0.5–287
- Mean ± SD	60.1 ± 57.2
Further treatment	
- Anti-androgen therapy	243
Tumor location	
- Total/PET/CT positive patients	660/500
Local recurrence	188 (28%/38%)
Metastases	397 (60%/79%)
- local/regional	203 (31%/41%)
- distant	98 (15%/20%)
- local and distant	96 (15%/19%)
Lymph node metastases	299 (45%/60%)
- local/regional	221 (33%/44%)
- distant	23 (3%/5%)
- local and distant	54 (8%/11%)
Bone metastases	182 (28%/36%)
Other metastases (i. e. lung, soft tissue)	29 (4%/6%)

PSA, prostate-specific antigen; vel, velocity; dt, doubling time; SD, standard deviation; n, number of patients; y, year.

**Figure 3:**
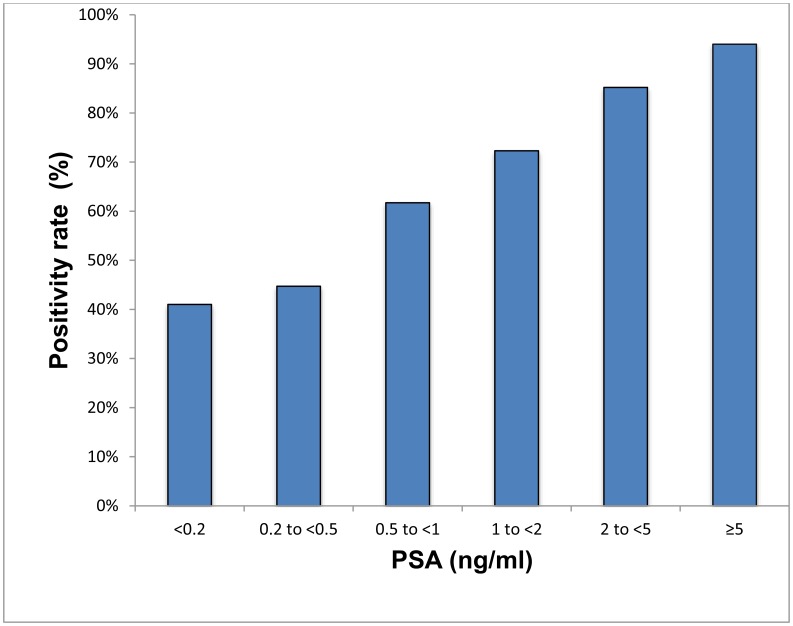
Positivity rate with respect to PSA.

The detection efficacy of 68Ga-PSMA-11 PET/CT was 41% (16), 45% (34), 62% (50), 72% (73), 85% (138) and 94% (189) for PSA levels <0.2 ng/ml, 0.2 to <0.5 ng/ml, 0.5 to <1 ng/ml, 1 to <2 ng/ml, 2 to <5 ng/ml, and ≥5 ng/ml respectively (*p* < 0.001) (Supplementary Table 1, Figure 3).

Local recurrence was evident in 38% of the patients with a positive PET/CT. Seventy-nine of the patients with a positive PET/CT showed metastases at different sites: 41% regional (local), 20% distant, 19% local and distant metastases, 20% single and 53% multiple metastases. Lymph node (LN) metastases were determined in 60%, of which 44% were regional, 5% distant, and 11% local and distant LN metastases; bone metastases were detected in 36% of the patients (Supplementary Table 1, Tables 1, 2).

**Table 2:** Location of PC in relation to PSA, PSAdt and PSAvel of patients with PC recurrence

	PSA (ng/ml)	*p* value	PSAvel (ng/ml/y)	*p* value	PSAdt (months)	*p* value
Number of patients	660		225		660	
AT	31.6 ± 149.6	*p* < 0.001	17.0 ± 72.9	*p* < 0.001	22.3 ± 35.6	*p* = 0.043
No AT	7.3 ± 36.1		3.4 ± 15.5		26.5 ± 60.6	
Local recurrence only	19.8 ± 146.6	*p* < 0.001	9.2 ± 24.5	*p* = 0.017	25.8 ± 47.2	*p* = 0.22 n. s.
No local recurrence	14.8 ± 65.6		9.6 ± 56.1		24.7 ± 54.9	
Metastases only	24.9 ± 122.7	*p* < 0.001	15.2 ± 65.2	*p* < 0.001	18.5 ± 30.4	*p* = 0.011
No metastases	3.2 ± 7.1		1.2 ± 2.3		34.8 ± 73.9	
LN metastases only	26.6 ± 138	*p* < 0.001	15.2 ± 67.6	*p* < 0.001	18.1 ± 30.1	*p* = 0.025
No LN metastases	7.7 ± 29.5		5.7 ± 34.8		30.7 ± 65.4	
Bone metastases only	40.4 ± 164.3	*p* < 0.001	14.9 ± 49.6	*p* < 0.001	17.9 ± 32.6	*p* = 0.002
No bone metastases	7.1 ± 46.1		6.9 ± 51.3		27.7 ± 58.5	

PSA, prostate-specific antigen; vel, velocity; dt, doubling time; AT, anti-androgen therapy; data are means and SD, standard deviation; *p* < 0.05 is considered significant.

Positive PET/CT scans were associated with local recurrence (0.357 *p* < 0.001), occurrence of metastases (0.695, *p* < 0.001), local/distant metastases (0.555, *p* < 0.001), AT (0.197, *p* < 0.001), single/multiple metastases (0.634, *p* < 0.001), local and distant LN metastases (0.415, *p* < 0.001) and bone metastases (0.349, *p* < 0.001) as well as GS (0.284, *p* < 0.001) for four and five-tiered GS groups.

Patients with a positive PET/CT finding showed significantly higher PSA levels and higher/faster PSAvel than patients without indicators for recurrence of PC. Patients with positive 68Ga-PSMA-11 PET/CT scans showed a shorter PSAdt, but this difference did not reach significance (*p* = 0.74) (Table 3, Supplementary Table 1).

**Table 3:** Differences between PET-positive and negative patients regarding PSA, PSAdt, PSAvel and MBq 68Ga-PSMA-11 application

	PET/CT positive	PET/CT negative	p value
Age (y) (660)	70.3 ± 7.2	70.6 ± 7.3	n. s.
PSA (ng/ml) (660)	20.76 ± 84.89	1.31 ± 1.75	*p* < 0.001
PSAvel (ng/ml/y) (225)	13.71 ± 61.35	0.85 ± 1.88	*p* < 0.001
PSAdt (months) (660)	19.49 ± 31.41	48.95 ± 111.9	n. s.
MBq (660)	193 ± 48	202 ± 50	n. s.

PSA, prostate-specific antigen; vel, velocity; dt, doubling time; MBq, applied amount of radioactive tracer 68Ga-PSMA-11; data are means and SD, standard deviation; *p* < 0.05 is considered significant.

**Figure 4:**
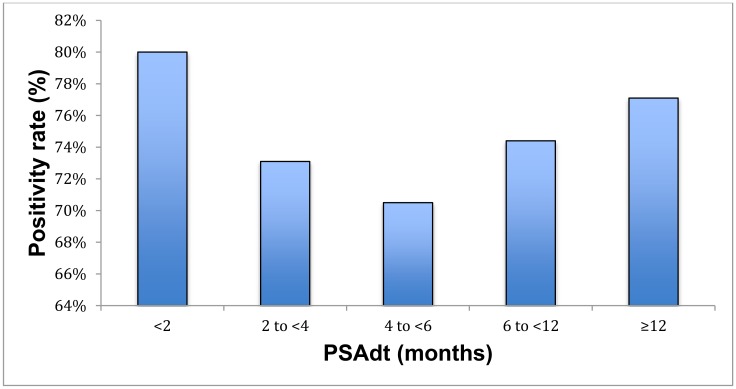
Positivity rate with respect to PSAdt.

The detection efficacy of PSAdt was 80%, 73.1%, 70.5%, 74.4% and 77.1% of patients with PSAdt of <2 months, 2 to <4 months, 4 to <6 months, 6 to <12 months and ≥12 months (*p* = 0.74) and 74.5%, 74.4% and 77.1% of patients with PSAdt of <6 months, 6 to <12 months and ≥12 months, respectively (Supplementary Table 1, Figure 4). PSAdt was associated with positive PET/CT results (r=0.027, *p* = 0.74 n. s.), with bone metastases (r=-0.073, n. s.) and local recurrence (r=0.073, n. s.). At short PSAdt (<6 months), intermediate (6 to <12 months) and longer PSAdt (≥12 months), 29%, 40% and 41% of patients were treated with AT, respectively.

**Figure 5:**
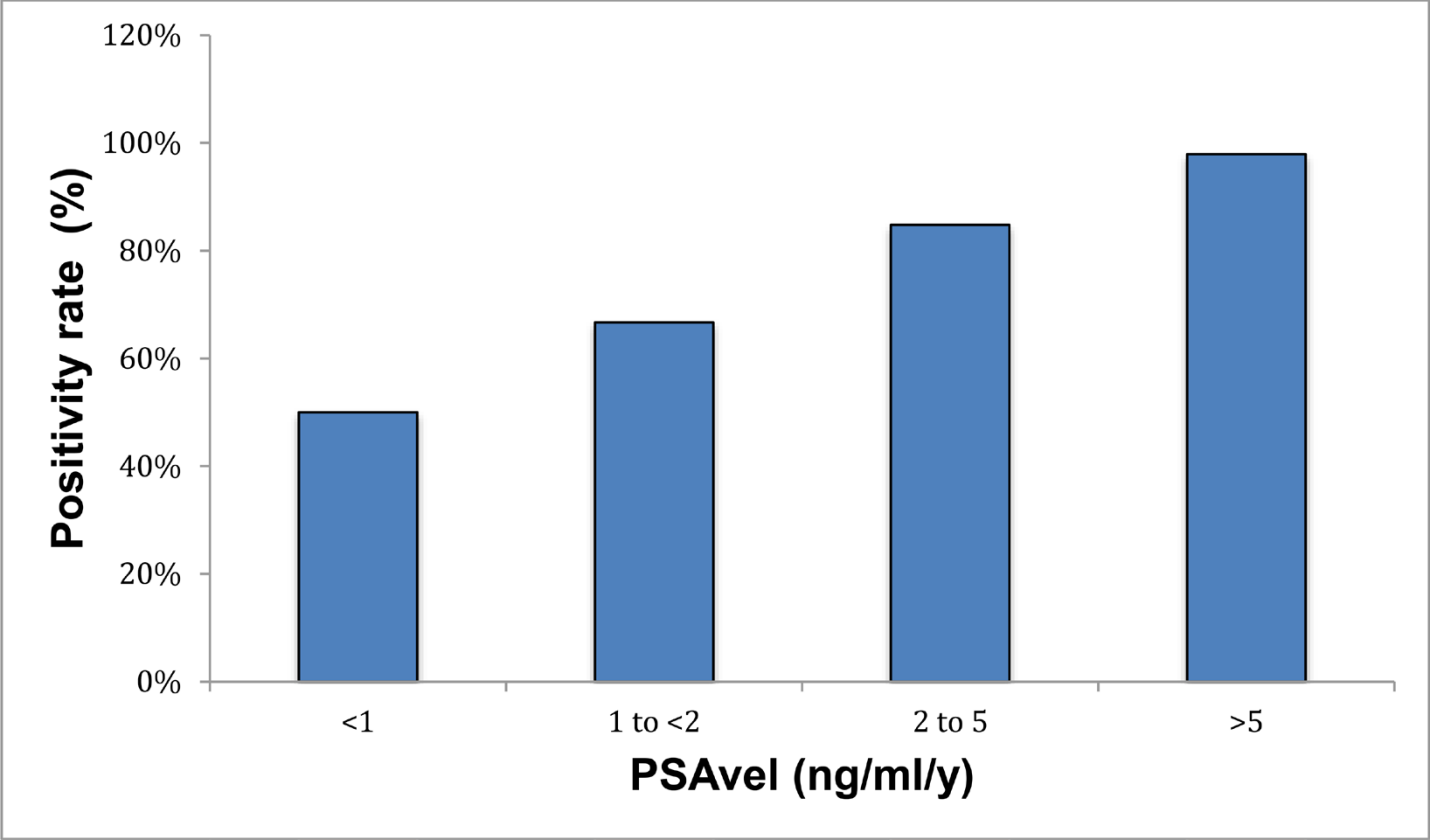
Positivity rate with respect to PSAvel.

The detection efficacy of PSAvel was 50%, 66.7%, 84.8% and 97.9% for PSAvel of <1 ng/ml/y, 1-<2 ng/ml/y, 2–5 ng/ml/y and >5 ng/ml/y, respectively (*p* < 0.001) (Supplementary Table 1, Figure 5). Seventeen percent of patients with PSAvel <1 ng/ml/y showed local recurrence and 42% exhibited metastases. Local recurrence was determined in 32% and metastases in 94% of the patients with high PSAvel >5 ng/ml/y (Supplementary Table 1).

Continuous PSAvel was different comparing patients with positive and negative scans (*p* < 0.001). However, categoric PSAvel correlated with positive PET/CT scans (0.421, *p* < 0.001), metastases (local and/or distant 0.403, *p* < 0.001), single/multiple metastases (0.453, *p* < 0.001), local and/or distant LN metastases (0.263, *p* = 0.005), local recurrence (0.173, *p* = 0.018) and bone metastases (0.267, *p* < 0.001).

Values of PSA, PSAdt and PSAvel in relation to sites of recurrence are shown in Table 2 and Supplementary Table 1.

### 68Ga-PSMA-11 PET/CT results compared to GS

Forty-four percent of patients with a positive 68Ga-PSMA-11 PET/CT scan were categorized GS<7, 53% showed a GS 7a, 80% a GS 7b, and 87% a GS>7.

Of the 45 patients with a GS<7 6%, 18%, 18%, 18%, 22% and 18% showed relapse PSA values <0.2 ng/ml, 0.2 to <0.5 ng/ml, 0.5 to <1 ng/ml, 1 to <2 ng/ml, 2 to <5 ng/ml and ≥5 ng/ml, respectively. The PET/CT results for GS<7 were positive in 44% of the patients, local recurrence and metastases were determined in 27% and 20%, respectively (Table 4). A minority of patients with GS<7 were treated with AT. Of 278 patients with GS>7, 87%, 31% and 76% showed positive PET/CT scans, local recurrence and metastases, respectively. Twenty-one percent and 40% of the patients with high GS>7 had PSA levels 2-<5 ng/ml and ≥5 ng/ml, respectively (Table 4). There was a significant correlation of GS and positive PET/CT (0.284 for four tiered GS, *p* < 0.001). Compared to patients with low GS, patients with GS>7 showed a high frequency of positive imaging (87% vs. 44%), of metastases (76% vs. 20%) and local recurrence (31 vs. 27%) (*p* < 0.001). Subgroups GS 7a and 7b also showed an increasing percentage of patients with positive PET/CT results (7a 53% vs. 7b 80%, *p* < 0.001), metastases (7a 36% vs. 7b 62%, *p* < 0.001), but not significant for local recurrence (7a 26% vs. 7b 27%, n. s.). Patients with GS 8 frequently showed a positive PET/CT result (91%), 76% exhibited metastases (multiple metastases 56%, LN metastases 61% which were mainly regional/local 42%), 30% showed bone metastases, and 31% exhibited local recurrence. Patients with GS 7a showed fewer multiple (7a 19% vs. 7b 44%) and distant metastases (7a 3% vs. 7b 15%) compared to patients with GS 7b (Table 4).

**Table 4:** Gleason Score in relation to localization of scan results and frequency, PSA, PSAdt and PSAvel

*n* = 660	GS < 7 (45)	GS 7a (115)	GS 7b (222)	GS 8 (118)	GS > 8 (160)	Chi^2^ r, *p* value
PET/CT positive	20	61	178	107	134	0.284; *p* < 0.001
AT	9	25	75	52	82	0.226; *p* < 0.001
Local recurrence	12	30	61	37	48	0.035; n. s.
Metastases	9	41	137	90	120	0.334; *p* < 0.001
Site of M						0.328; *p* < 0.001
Local M	4	35	70	39	55	
Distant M	3	4	34	24	33	
Local and	2	2	33	27	32	
Distant M						
Number of M						0.309; *p* < 0.001
Single M	4	19	40	24	45	
Multiple M	5	22	97	66	75	
Lymph node M	7	33	106	72	81	0.204; *p* < 0.001
Site of LN M						0.200; *p* < 0.001
Local	4	32	79	50	57	
Distant	1	1	5	10	6	
Local and distant	2	0	22	12	18	
Bone M	2	8	59	35	78	0.321; *p* < 0.001
Other M (i. e. lung, soft tissue)	1	2	5	14	7	0.084; *p* < 0.001
**PSA (ng/ml)**						0.125; *p* = 0.005
< 0.2	3	9	10	11	6	
0.2 to <0.5	8	19	21	9	19	
0.5 to <1	8	11	31	15	16	
1 to < 2	8	17	43	10	23	
2 to <5	10	36	57	23	36	
≥ 5	8	23	60	50	60	
**PSAdt (month)**						*p* = −0.096; n. s.
< 6	8	35	67	34	60	
6 to <12	7	22	46	21	33	
≥ 12	30	58	109	63	67	

*n* = 225	GS < 7(16)	GS 7a(40)	GS 7b(61)	GS 8(30)	GS > 8(78)	Chi^2^ r, *p* value
**PSAvel (ng/ml/y)**						0.207; n. s.
< 1	13	24	29	12	34	
1 to <2	0	8	11	3	11	
2 to 5	2	5	7	7	12	
>5	1	3	14	8	2	

LN, lymph node; M, metastases; (*n*), number of patients; data are percentage of patients; *p* < 0.05 is considered significant; r, Pearson correlation coefficient, PSA, prostate-specific antigen; PSAdt/vel, PSA doubling time/velocity; AT, anti-androgen therapy.

There were significant differences between relapse PSA levels of GS 7a vs. GS >7 as well as GS 7a and 7b (*p* = 0.005). No significant association was found for PSAdt and PSAvel (Table 4).

GS groups (four-tiered in brackets) were significantly associated with PET/CT results (0.284, *p* < 0.001), with, metastases (0.334, *p* < 0.001), and local and distant metastases (0.328, *p* < 0.001), single and multiple metastases (0.309, *p* < 0.001), LN metastases (0.204, *p* < 0.001), local and/or distant LN metastases (0.200, *p* < 0.001), bone (0.321, *p* < 0.001), and other organ metastases (0.084, *p* < 0.001), but not with local recurrence (0.035, n. s.) (Table 4).

### Influence of anti-androgen therapy

Out of all patients, AT was reported in 37% (243) of the patients (Table 1). 87% (211) patients, that were AT-treated, showed a positive PET/CT. AT-treated patients showed significantly higher relapse PSA levels than patients not treated with AT (31.6 ± 149.6 ng/ml vs. 7.3 ± 36.1 ng/ml, *p* < 0.001) and significantly higher PSAvel (17 ± 72.9 ng/ml/y vs. 3.4 ± 15.5 ng/ml/y, *p* < 0.001), whereas PSAdt was not comparable (22.3 ± 35.6 months vs. 26.5 ± 60.6 months, *p* = 0.043), but also significant (Table 2).

AT was associated with PET/CT results (0.197, *p* < 0.001), relapse PSAV (0.240, *p* < 0.001), PSAvel (0.332, *p* < 0.001), PSAdt (0.108, *p* = 0.019), occurrence of metastases (0.198, *p* < 0.001), GS (5-tiered 0.226, *p* < 0.001), local and distant metastases (0.225, *p* < 0.001), bone metastases (0.218, *p* < 0.001) and single/multiple metastases (0.219, *p* < 0.001). AT was not correlated with local recurrence in the prostate bed (*p* = 0.62). However, there was an association of AT with all types of LN metastases (*p* = 0.01) and with the specific locations of LN metastases (local, distant and local with distant LN metastases) (*p* < 0.001).

High PSAV and high PSAvel were associated with a higher percentage of AT-treated patients (60% PSA ≥ 5 ng/ml vs. 31% <0.2; 74% PSAvel >5 ng/ml/y vs. 33% <1 ng/ml/y) (Supplementary Table 1). Four-tiered GS were associated with AT, 20% of the patients with GS<7 were treated with AT vs. 48% patients with GS>7 and 30% with GS=7 (GS 7a 22%, 7b 34%) (Table 4).

## DISCUSSION

Different 68Ga-labelled PSMA inhibitors have been studied regarding their sensitivity and specificity for the diagnosis of recurrent PC; e. g., 68Ga-labelled HBED-CC, which is an efficient 68Ga-chelator [13]. Eder *et al*. showed, that the PET-imaging ability of an urea-based PSMA-inhibitor could be improved significantly with HBED-CC [14]. We therefore retrospectively analyzed the PR of restaging 68Ga-PSMA-11 PET/CT in a large cohort of patients (660) with BCR in relation to PSA levels, PSAdt, PSAvel and GS, taking AT into account.

### PSA and PR of 68Ga-PSMA-11 PET/CT

The PR of 68Ga-PSMA-11 PET/CT in the present study was 76% of all patients (mean PSA 20.76±84.89 ng/ml), confirming the results of previous studies in which detection rates (DR) of 74% to 81% were reported for restaging PET/CT. The relationship of PSA, PSAdt, GS and 68Ga-PSMA-11 PET/CT was recently analyzed in patients with PC recurrence. 68Ga-PSMA-11 PET/CT was positive in most patients with PSA levels of at least 2 ng/ml, in 80% of patients with PSA between 1–2 ng/ml and even at PSA levels ≤1 ng/ml, and a clinically relevant percentage of patients (44%) were 68Ga-PSMA-11 PET/CT positive [1, 15–17]. We demonstrate here, that the PR declined with decreasing PSAV. At PSA levels from 2 ng/ml and above 5 ng/ml, the PR was 85.2% and 94% ng/ml, respectively, which also corroborates the results of other investigators [16]. We further showed that even at PSA levels below 0.5 ng/ml, the PR was between 41% (PSAV <0.2 ng/ml) and 44.7% (PSAV 0.2 to <0.5 ng/ml). For PSAV of 0.5 to <1 ng/ml the PR was 61.7%. These data corroborate the findings of recent studies of restaging by 68Ga-PSMA PET/CT where the DR with PSA levels of 0.2–0.49 ng/ml was still 50%, and in a different study, 53% of the patients were 68Ga-PSMA PET/CT positive at restaging PSA levels of 0.5–0.99 ng/ml [1]. Other investigators also showed, that in almost 70% of patients with PSA levels below 1 ng/ml, the site of recurrence could be identified using 68Ga-PSMA PET/CT [16]. The results from the present study underline the conclusion that 68Ga-PSMA-11 PET/CT is a sensitive tool for restaging recurrent PC.

### PSA and DR of other radioligands

Other radioligands such as 11C- and 18F-choline PET/CT exhibit lower DR at low PSA levels than 68Ga-PSMA PET/CT. A linear correlation was observed between PSA and 11C-choline positive scans [8, 18–20]. 11C-choline detected recurrence in 73% of the patients with PSAV of at least 3 ng/ml, but with PSAV under 1 ng/ml the DR decreased to 19–36% [3, 21]. Likewise, the DR in patients with a small increase of PSA (<1.5 ng/ml) was only 28% using 11C-choline PET/CT [3]. The DR of 68Ga-PSMA PET/CT is also higher than that of 18F-choline PET/CT [21, 22]. The optimal PSA threshold was shown to be 1.7 ng/ml for 18F-choline and 1.4 ng/ml for 11C-choline PET/CT [18, 21]. However, 18F-labelled PSMA radiotracers are a highly promising alternative to 68Ga-labelled compounds while offering some important advantages, e. g. longer half-life, and potentially (not proven in a prospective trial) improved lesion detectability. A recent study demonstrated that the second-generation radiofluorinated PSMA (18F-DCFPyL-PSMA) showed a high DR at low PSA levels with 88% [23, 24].

### PSA, PSAvel and tumor location

In recurrent PC we could demonstrate that PSA was significantly higher in PET-positive vs. PET-negative scans and that PSA was correlated with local and systemic recurrence. Patients with bone metastases showed higher baseline PSA than patients with local recurrence or LN metastases only (median of 5.1 ng/ml vs. 3.7 ng/ml and 2.4 ng/ml, respectively). PSAvel ranged from 1.33 ng/ml/y in patients with LN metastases only, to 2.65 and 3.31 ng/ml/y if patients had local recurrence or bone metastases only. PSAdt did not differ among most tumor locations. There was no association of PSAdt with 68Ga-PSMA-11 PET/CT PR (75–77%), local recurrence (25–32%) and bone metastases (29–23%) and it was not associated with the degree of disease (multiple distant metastases) (Supplementary Table 1). Likewise, Eiber *et al*. also did not observe an association between DR and PSAdt [16].

PSA and PSAvel were significantly higher in PET/CT positive patients, compared to PET/CT negative patients (PSA: 20.76 vs. 1.31 ng/ml; PSAvel: 13.71 vs. 0.85 ng/ml/y) (Table 3). PSAdt also differed between PET/CT positive and negative patients (PSAdt: 19.49 vs. 48.95 months), but without significance. The PR of metastases and local recurrence increased with increasing PSAvel (50% at <1 ng/ml/y and 97.9% at >5 ng/ml/y) (Supplementary Table 1). The PR at low PSAvel in our study was lower than that reported by Eiber *et al*. (2015) who observed a DR of 82% at PSAvel of <1 ng/ml/y [16]. Verburg *et al*. (2016) reported that PSAV ≥2 ng/ml were associated with high DR for local PC, extrapelvic LN, bone and visceral metastases. Short PSAdt was associated with pelvic and extrapelvic LN metastases as well as with bone and visceral metastases. The authors postulated PSAdt as an independent determinant of bone metastases and both PSA and PSAdt as determinants of positive scans and extrapelvic LN metastases [15]. We also observed significant differences of continuous PSAV and PSAvel (*p* < 0.001) between PET/CT positive vs. negative patients, but no significance was found for PSAdt (Table 3). We found an association of positive scan results with tiered PSAV (0.442, *p* < 0.001) and PSAvel (0.421, *p* < 0.001), but not with tiered PSAdt (0.027, *p* = 0.74) (Tables 3, Supplementary Table 1). Likewise, Verburg *et al*. (2016) demonstrated that high PSA levels correlate with positive 68Ga-PSMA PET/CT scans and with short PSAdt. The results of our study and those of other investigators demonstrate, that 68Ga-PSMA-11 PET/CT is a sensitive tool for the detection of PC recurrence in patients even with low PSA levels [15].

In addition, we showed a high correlation of PSAV with PSAvel (Spearman-Rho, r: 0.852, *p* < 0.001).

We observed an increasing degree of disease in patients with high baseline PSAV and high PSAvel. With increasing PSAvel, we observed a marked increase of the frequency of local recurrence and of metastases, mainly distant metastases (9% vs. 32%), a four times higher frequency of multiple metastases (17% vs. 66%) and a doubling of the frequency of LN metastases (29% vs. 60%) (Supplementary Table 1). In comparison, Graute *et al*. (2012) showed that patients with metastases in two or more organs exhibited high baseline PSA and high PSA kinetics [18].

### Comparison with the results of other radioligands

As compared to other radioligands, for positive 18F-choline PET/CT, PSAdt of 6.7 months was detected and in PET-negative patients, PSAdt was 9.3 months. The optimal PSAdt cut-off for the detection of recurrent PC using 18F-fluorocholine was 3.2 months [18, 20]. For 11C-choline PET/CT, the cut-off was 7.2 months. The DR by 11C-choline PET/CT ranged from 40% in patients with high PSAdt and increased to 60% in patients with fast PSAdt. Similarly, more positive 11C-choline PET/CT scans were observed in patients with high PSAvel. In addition, patients with short PSAdt exhibited a higher degree of disease with distant metastases, whereas a long PSAdt was associated with local recurrence [3].

### Applied amount of radioactive tracer 68Ga-PSMA-11

In the present study, the injected amount of radioactive tracer was not different comparing PET-positive and negative patients. This corroborates the findings of a recent investigation with 68Ga-PSMA-11 [17].

### GS

In our study, high PR was associated with high GS. Patients with GS of 7a, 7b and GS of 8 showed a PR of 53%, 80% and 91%, respectively vs. 44% in patients with GS<7. In addition, the degree of disease increased with increasing GS. Multiple metastases occurred in 35%, 44% and 56% of patients with GS=7, 7b and 8 vs. 19% and 11% in patients with GS 7a and GS<7, respectively. Metastases, local and distant metastases and local LN metastases were three to five times as frequent, bone metastases were more than seven times as frequent, comparing patients with high and low GS (Table 4).

GS correlated significantly with PET/CT results (*p* < 0.001) and PSAV (*p* = 0.005). Patients with GS 7b and >7 frequently showed a higher, e. g. PSA ≥5 ng/ml and a higher PSAvel, e. g. >5 ng/ml/y. PSAV (subgroup ≥5 ng/ml) and PSAvel (subgroup >5 ng/ml/y) of patients with GS 7b and over 7 were tendencially higher than those of patients with lower GS (7a or <7) (Table 4). In another study, the DR for 68Ga-PSMA PET/CT was also significantly higher in patients with a GS of at least 8 than that of patients with a GS of 7 and lower (87% vs. 97%, respectively) [16]. Similarly, patients with a GS of at least 7 were shown to have shorter BCR-free probability [25]. This is not in line with the results of other investigators, who demonstrated that tumor DR was not associated with GS. Only in patients with PSA <0.2 ng/ml, GS was reported to be significantly higher in PET-positive patients, whereas log PSA value was equal [17].

### AT

In our study, AT was associated with positive 68Ga-PSMA-11 PET/CT results and degree of disease. Especially patients with high PSAV, high PSAvel and high GS were treated with AT. PSAV, PSAvel and PSAdt of patients treated with AT were significantly higher respectively vs. patients that were not treated. This corroborates the results of a recent study, where patients treated with AT showed positive PET/CT results more often, which was thought to be due to the frequent use of AT in patients with advanced disease [8, 16, 17]. Considering the degree of disease, about half of the patients with both local and distant metastases as well as multiple metastases were treated with AT. The correlation that patients with faster PSAdt are more likely to receive AT, and more likely to be those with metastatic disease, indicates a new trend in hormone therapy. Our results underline that the current selection of patients, treated with AT, has improved significantly in the current decade. That is, the more appropriate patients are now actually treated with hormone therapy, and not those with localized cancer without metastases as well as those with a slow doubling time. Pagliarulo *et al*. showed that patients with metastastic PC have a significant advantage of AT with respect to quality of life and reduction of disease-related morbidity. On the other hand, no significant benefit could be shown in men with non-metastatic disease [26–28].

In our study, AT was performed in 51% of the patients with GS >8 and only 20% in patients GS <7. While 22% patients with GS 7a, 34% with GS 7b and 44% with GS 8 were treated with AT.

### Management change

Recent studies could show that 68Ga-PSMA-11 PET/CT results can change initial TNM staging and re-staging in the setting of BCR, leading to an adjustment of radiotherapy management in PC patients, which was also corroborated with our own experience [29]. According to Sterzing *et al*., radiotherapeutic management was changed in 51% of the patients after restaging with 68Ga-PSMA PET/CT-11 which suggests the possibility of selective and individualized therapeutic decision-making in patients with PC recurrence [30]. In the setting of advanced prostatic cancer disease and castration-resistant metastatic PC, PSMA PET/CT plays an important role in targeting 177Lutetium-PSMA radioligand therapy and treatment response assessment [31].

## MATERIALS AND METHODS

### Patients

We retrospectively studied 68Ga-PSMA-11 PET/CT (Glu-urea-Lys(Ahx)-HBED-CC) scans of 660 patients with BCR of PC (07/2015 until 01/2019) that had previously been treated for localized cancer. BCR was defined as an increase of serum PSA levels after first treatment of at least 0.2 ng/ml in three consecutive samples or >2 ng/ml above nadir after RT. Additionally 39 patients with three consecutive PSA elevations <0.2 ng/ml were included, although they didn’t literarily belong to the BCR definition and we have defined them as a special but clinically very interesting subgroup. The time between primary treatment and 68Ga-PSMA-11 PET/CT was 60.1±57.2 months (median 41, range 0.5–287 months). PSA levels were available at the time of surgery, nadir, interim and at the time of PET/CT (relapse). Mean relapse PSA levels were 2.46 ng/ml ± 95,8 ng/ml (median 10.65, range 0.1–2000). Two hundred forty-three patients were treated with AT (Table 1).

The patient data and scans were collected from four Nuclear Medicine Departments (NMDa, NMDb, NMDc, NMDd) in Germany.

### Patients were divided into groups:

Six PSA groups: <0.2 ng/ml, 0.2 to <0.5 ng/ml, 0.5 to <1 ng/ml, 1 to <2 ng/ml, 2 to <5 ng/ml as well as ≥5 ng/ml. Three PSAdt groups: <6 months, 6 to <12 months and ≥12 months and four PSAvel groups: <1 ng/ml/y, 1 to <2 ng/ml/y, 2–5 ng/ml/y and >5 ng/ml/y.

Furthermore, patients were assigned to four and five-tiered GS groups: <7, =7 and >7 (GS 8, 9) (four-tiered GS) and <7, 7a and 7b, >7 (GS 8, 9) (five-tiered GS).

Our study was in accordance with the Helsinki Declaration and the German Medicinal Products Act, AMG §13.2b and all patients gave their written informed consent. The retrospective study was approved by the ethics committee.

### Radioactive labelling

The general procedure for the labelling of the HBED-CC conjugate (PSMA-specific pharmacophore Glu-NH-CO-NH-Lys) with 68Ga, is based on the slightly modified process described by Eder *et al*. [14]. Briefly, 68Ga generator (Garching, Germany) eluate (0.05 M HCl, 1.2 ml) was reacted with a 430 μl aqueous solution containing 26.4 μM precursor (CAS no. 1366302-52-4; ABX, Radeberg, Germany) PSMA-11 in 0.28 M NH_4_OAc for 1 min at 30 °C. The labelled no-carrier-added tracer was purified via a reverse-phase cartridge (Sep-Pak C18 Plus Light cartridge, 130 mg Sorbent; Waters) and formulated in 10 ml phosphate-buffered saline containing 5 vol% EtOH. Radiochemical purity (RCP) was >98% as confirmed by thin layer chromatography (TLC) and high performance liquid chromatography (HPLC).

68Ga-PSMA-11 was obtained from the Department of Nuclear Medicine of the University of Mainz (NMDa-NMDc), from Eckert & Ziegler Bonn (NMDc) or Advanced Accelerator Applications Bonn (NMDb, NMDd).

### Quality control

To determinate the incorporation, instant TLC (ITLC) on ITLC-salicylic acid (SA) impregnated glass fiber sheet from Varian was performed. 5 μl sample of the final fraction was spotted on a SA impregnated glass fiber sheet as stationary phase and a solution mixture of ammonium acetate 1M/methanol 1:1 as mobile phase. The free Ga-68 remains at the start position and the Ga-68 labelled PSMA-11 migrates with the solvent front. The scan was recorded with Gina Star TLC and analyzed by miniGita software from Raytest (Straubenhardt, Germany) as well as by the cutting method with measuring the single sections. RCP is defined as the % of activity of the radionuclide, present in the desired radiopharmaceutical form of the total radioactivity.

As a second method to determinate the RCP, Radio-HPLC measurements using the Dionex HPLC 3000 system (Idstein, Germany) with a Perfect Bond ODS-H 5μ column (4.0 9 100 mm, MZ-Analysentechnik, Mainz) and a CH_3_CN/H_2_O/0.1% TFA linear gradient (from 20 to 100% in 8 min) as mobile phase at a rate of 2.6 ml/min was performed.

### Imaging procedure

Whole body 68Ga-PSMA-11 PET/CT was performed about 60 min. after intravenous injection of 68Ga-PSMA-11 (mean and SD: 198±61 MBq, range 50–390 MBq, median 199 MBq). PET/CT scans were acquired using a Gemini TF16 PET/CT scanner (Philips Medical Systems, Best, Netherlands) with transverse and axial field of views of 57.6 cm and 18 cm, respectively (NMDa, NMDd) or a Biograph 64/Z64, R4 (HD+Time of Flight)/ Biograph 64 TruePoint (True V HD) PET/CT scanner (Siemens, Erlangen, Germany) (NMDb, NMDc). Patients were scanned in caudo-to-cranial direction with raised arms. Either only a mid-inspiratory low-dose CT scan without contrast medium (20–60 mAs, 120 kV, CT transverse scan field 50 cm, 70 cm extended field of view, high contrast-resolution 1.0 s, 0.6 mm) or in addition, a maximum inspiratory venous phase diagnostic contrast enhanced CT scan (100–400 mAs, 140 kV, dose modulation) from the base of the skull to the upper thigh was performed for attenuation correction and anatomical correlation. Immediately thereafter, a PET scan of the same body regions was performed (NMDb-NMDc). Hospital NMDa performed scans with low dose CT only, without contrast media. Scans started 70 s after intravenous injection of contrast medium (Ultravist 300; Bayer AG, Berlin, Germany; body surface area adapted volume of 74.2 ml/m^2^, 22.26 g I/m^2^). CT reconstruction was performed with a medium-smooth soft-tissue kernel (window centre 60, width 450) at a slice thickness of 5 mm with an overlapping increment of 3.5 mm. In indeterminate cases late scans 3 hrs p. i. with a urographic low dose scan of the abdomen, and facilitation of urinary excretion with hydratation and furosemide i. v. were added.

Immediately after the CT scan, the PET scan of the same body part was performed. PET scans were acquired in 3D with an acquisition time of 3 minutes per bed position (axial field view of NMDa: 18 cm, NMDb: 21.8 cm, NMDc: 13.3 cm, NMDd: 19 cm). Random, scatter and decay correction were applied to the emission data. CT data were converted to attenuation coefficients at 511 keV and used for attenuation correction. An ordered-subsets expectation maximization (OSEM) algorithm was used for reconstruction (NMDc) matrix: 400 × 400, three iterations, 24 subsets, postreconstruction smoothing Gaussian filtering, 5 mm transaxial resolution, full-width at half maximum, NMDb, NMDd: matrix: 168 × 168 (NMDb), 144 × 144 (NMDd), two iterations, fourteen subsets, Gaussian filtering, 4.2 mm (NMDb) or 5 mm (NMDd) transaxial resolution, full-width at half-maximum; NMDa: matrix: 144 × 144, 3 iterations 33 subsets, Gaussian filtering 4.3 mm full-width at half-maximum.

### Image analysis

The uptake of 68Ga-PSMA-11, i. e. the tracer concentration of the hypermetabolic lesion detected in the image, was quantified in terms of SUV_max_. Disease recurrence was defined as a PSMA-avid lesion, corresponding to a morphological substrate on CT imaging. All images were assessed in a consensus reading by at least one experienced board-certified nuclear medicine physician, and two experienced board-certified radiologists, each with more than 5 years of PET/CT experience and extensive experience with 68Ga-PSMA PET/CT. The assessing physicians scored scans regarding the presence of lesions in the prostate or prostate bed and/or seminal vesicles as well as with regard to the presence of LN metastases in the pelvis or extrapelvic LN (N1 vs. M1a disease) in accordance with the 7th edition of the Union International Contre le Cancer Tumour, Node, Metastases system [32]. The presence of bone metastases and the presence of visceral metastases were also scored. When conflicting interpretations were identified, they were discussed and solved by the panel and consensus was passed between the reviewing physicians. Additionally, structured Reports in PK were generated using a software reporting system (Mint lesion®, Mint Medical GmbH, Dossenheim), to allow for a TNM classification of the findings.

### Data analysis

For analysis, patients were divided into six groups according to their (PSAV) at relapse examination, PSAdt, PSAvel and four and five-tiered GS: PSAdt was calculated based on available PSAV according to other authors [16] and PSAvel according to Graute *et. al*. [18].

The following parameters were determined: 68Ga-PSMA-11 PET/CT results, PSA (at the time of treatment of PC, nadir, interim and relapse, when PET/CT was performed), local/regional, distant, single, multiple metastases, LN or bone metastases, other sites of metastases (e. g. lung), and GS as well as AT. Because histopathology, as the true gold standard, was available only in a minority of patients, a true detection rate could not be verified.

### Statistical analysis

Statistical analyses were performed using SPSS version 23.0 (IBM Corporation, Ehningen, Germany). Most of the metric variables were not normally distributed, as assessed by Kolmogorv-Smirnov or Shapiro-Wilk test. To determine differences between two groups (PET/CT positive vs. negative results), for normally distributed metric variables (e. g. age, injected activity), independent samples *t*-tests were performed and for not normally distributed variables (PSAdt, PSAvel) Mann Whitney *U*–Test, respectively. Wilcoxon signed rank test was calculated to determine changes of PSA levels. Additionally for further analysis, metric values (e. g. PSAV, PSAdt and PSAvel) were split into categories. These nominal and ordinal parameters were analyzed with Chi-square tests, Fisher exact tests and Pearson correlation. Data are presented as means, standard deviation and/or median and range. *P* < 0.05 was considered significant.

## CONCLUSIONS

In the present study we demonstrate the sensitive detection of PC recurrence and PC metastases using 68Ga-PSMA-11 PET/CT in a large cohort of patients. Even at low PSA levels under 0.2 ng/ml, the PR was 41%, which shows that 68Ga-PSMA-11 PET/CT can detect PC recurrence early, therefore enabling individualized therapeutic decision-making and risk adapted treatment as well as selecting suitable patients for radionuclide therapy with 177Lutetium-PSMA.

## SUPPLEMENTARY MATERIALS




