# Supplementary material for: The positivity rate of 68Gallium-PSMA-11 ligand PET/CT depends on the serum PSA-value in patients with biochemical recurrence of prostate cancer
Source: Oncotarget. 2019 Oct 22;10(58):6124–37. doi: 10.18632/oncotarget.27239 (PMC6817454; doi:10.18632/oncotarget.27239)
Supplement: Supplementary file 1 [file oncotarget-10-6124-s001.pdf]

The positivity rate of 68Gallium-PSMA-11 ligand PET/CT depends on the serum PSA-value in patients with biochemical recurrence of prostate cancer

SUPPLEMENTARY MATERIALS

Supplementary Table 1: Location of PC recurrence with respect to PSA, PSAdt and PSAvel. See Supplementary Table 1
