# Supplementary material for: The positivity rate of 68Gallium-PSMA-11 ligand PET/CT depends on the serum PSA-value in patients with biochemical recurrence of prostate cancer
Source: Oncotarget. 2019 Oct 22;10(58):6124–37. doi: 10.18632/oncotarget.27239 (PMC6817454; doi:10.18632/oncotarget.27239)
Supplement: Supplementary file 2 [file oncotarget-10-6124-s002.docx]

**Supplementary Table 1: Location of PC recurrence with respect to PSA, PSAdt and PSAvel**

* Fisher exact test

PSA, prostate-specific antigen; vel, velocity; dt, doubling time; th., therapy; M, metastases; LN, lymph node; data are percentage of patients; means and SD; p<0.05 is considered significant; r, Pearson correlation coefficient
